# How Might Consideration of Cell Polarity Affect Daily Therapeutic Practices?A Literature Review -

**DOI:** 10.31661/gmj.v12i.2970

**Published:** 2023-05-28

**Authors:** Hamid Reza Ravanbod

**Affiliations:** ^1^ Podiatric Surgery, Australian Podiatry Association, Melbourne, Australia

**Keywords:** Bioelectricity, Cell Polarity, Bioelectric, Action Potential

## Abstract

Background: In addition to biochemical gradients and transcriptional networks, cell behaviour is controlled by endogenous bioelectrical signals resulting from the action of ion channels and pumps. Cells are regulated not only by their own membrane resting potential (Vmem) but also by the Vmem of neighbouring cells, establishing networks through electrical synapses known as gap junctions. V mem is the primary factor in producing a polarity that can regulate cell assimilation of various substances. This article aimed to examine how cell polarity can change and how variations in cell polarity may lead to clinical demonstrations.Materials and Methods: Using Cochrane Central, PubMed, Scopus, Web of Science (WOS), and Embase, a comprehensive qualitative literature review was conducted from February 1, 2018, to February 1, 2023, to identify studies addressing bioelectric, cell polarity, and electroceuticals in patients with foot and ankle problems.Results: Out of 1,281 publications, 27 were included. One study investigated bioelectric wound-healing. Twenty-five studies examined bioelectric nerve cell growth, whereas one study evaluated bioelectricity-induced cellular differentiation in the treatment of arteriopathies.Conclusion: The author of this systematic review support addressing the predisposing factors and healing impediments for a disease, thereby enhancing the healing process and reducing the likelihood of recurrence or parallel conditions. This method of treatment has provided a summary of evidence indicating that cell polarity could be addressed for the treatment and prevention of most if not all, foot and ankle problems. However, owing to the limitations of V mem and bioelectricity measurement and the direct or indirect involvement of genetics and chemical gradients, further studies are required to confirm these results.

## Introduction

Bioelectricity is an electrical phenomenon that is induced or supplied to cells to affect their phenotype. In the context of this term, an electrical phenomenon refers to functions or activities in living cells that depend on the separation of charges (voltage), which generally occurs when ions separate, or move (current), typically via channels and pumps. This electrical effect is generated by a living cell that expends energy to accomplish this. In other words, the dead cells do not generate bioelectricity. However, they can change the quality and quntity of bioelectricity in the adjacent cells. A "cell" according to this definition could be a single cell or a group of cells. A cell’s phenotype consists of its shape, size, location of charges in space and time, physiology, and gene expression. The phenotype covers the condition of the cell, including polarisation. Polarisation is the disruption of a region’s charge distribution by an electric field (EF) [1][2][3]. Evidence demonstrates that bioelectrical changes are fundamental to cellular differentiation and wound-healing [4]. 

Additionally, the application of physiological strength and transepithelial EFs can alter nerve cell migration, orientation, and growth [5]. However, the benefits of bioelectricity may extend well beyond these three uses. For instance, breakthroughs in molecular-level techniques [1] have identified a unique property of bioelectricity that governs the activity of single cells and coordinates the cellular development and regenerative repair of complex structures. This indicates that changes in bioelectricity and cell polarity can alter cellular programming, resulting in the development of new symptoms or the disappearance of undesirable symptoms. This review aims to address some of the well-known or commonly accepted uses of bioelectricity and cell polarity in the pathophysiology and treatment of foot and ankle disorders. In addition, it provides new perspectives on the potential of bioelectricity and cell polarity to prevent diseases and improve the treatment of foot and ankle disorders [6].

## Materials and Methods

*Protocol and Registration*

The protocol was developed according to the Preferred Reporting Items for Systematic Reviews and Meta-Analysis (PRISMA) Checklist [7] and was retrospectively registered on 08/02/2023 with the Open Science Framework (Registration number: osf.io/ehbtk). 

*Eligibility Criteria*

A comprehensive literature review was conducted using various databases including Cochrane Central, PubMed, Scopus, Web of Science (WOS), and Embase from February 1, 2018, to February 1, 2023. To identify additional studies, Google Scholar was also used. Key search terms and their synonyms, including bioelectric, cell polarity, electroceuticals, and foot and ankle diseases were combined using Boolean operators (AND, OR, and *). If full articles were not available or information was missing, we contacted the corresponding authors to request additional data. The review excluded papers that did not focus on the treatment of diseases that addressed non-human specimens or diseases other than foot and ankle. 

In addition, lecture notes, dissertations, and papers not published in peer-reviewed journals have been removed due to accessibility issues. Finally, the eligible papers were sorted into three main topics for evaluation: a) Wound-healing, b) Nerve healing, and c) Cellular differentiation and the reparative regeneration of complex structures.

## Results

*Study Selection*

**Figure-1:**
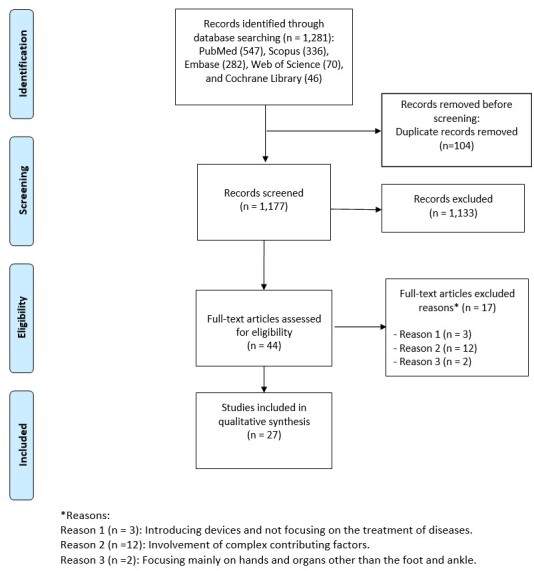
PRISMA flow diagram

We retrieved 1,281 studies from electronic databases and excluded 104 duplicate studies. Reading titles and abstracts, 1,133 papers were discarded, and the remaining 44 full-text articles were retrieved for potential inclusion. The author assessed citations, abstracts, and full-text publications based on the inclusion criteria. From these 44 articles, 17 studies were removed for various reasons, such as discussing devices instead of disease treatment (three articles), including complicated contributing factors (12 articles), or focusing only on hands and organs other than foot and ankle (two articles). The study selection process is shown in the PRISMA diagram (Figure-1)

*Characteristics of the Included Studies*

Eligible studies with different designs were grouped into three categories:

a) One study focused on wound-healing [8].

b) A total of 25 studies examined transepithelial EFs and their influence on cell migration, orientation, and nerve growth [9][10][11][12][13][14][15][16][17][18][19][20][21][22][23][24][25][26][27][28]. 

c) Another study explored the aspect of bioelectricity that regulate cellular differentiation and diabetic arteriopathy [19].

*Description of Studies and Their Characteristics*

A total of 1,281 citations were retrieved through a database search, and 27 studies were identified that addressed the topics of this analysis. Of these studies, three showed no statistically significant differences between the use of electric stimulators (ES) and conventional approaches [25][12][29]. These studies focused on reducing muscular spasms following a stroke or multiple sclerosis (MS).

The remaining 24 trials revealed a statistically significant effect of ES on symptom relief, but the studies considered possible changes in the bioelectricity or examined bioelectrical changes to determine device efficacy.

*Intervention Types Employed*

The available evidence about bioelectricity were analysed using descriptive qualitative analysis and coded by the author. As a result, three overarching categories were identified:

*Category 1: Wound-Healing*

Research indicates that bioelectrical signalling is a crucial aspect of the wound-healing process [8]. Understanding and effectively using this process is essential for improved wound-healing and regeneration. During this process, individual cell behaviours coordinate migration to the wound centre to address mild or severe barrier deficiencies [30]. One of the most critical processes involved in restoring the skin barrier is the migration of epithelial cells as a continuous sheet structure. When skin is injured, weakened epithelial barriers create endogenous EFs, sustained by ion channels and cell junctions, with the cathodal pole at the centre of the incision [31]. In response to electrical impulses, epithelial cells recognize minute EFs and move in a designated direction [32]. It has long been hypothesised that naturally occurring EFs promote wound-healing by directing cell migration [33], and experimentally gathered evidence indicates that extensive epithelial sheets of keratinocytes or corneal epithelial cells collectively respond to applied EFs [8]. Although some mechanisms of collective cell migration are similar to those employed by solitary cells, the coordinated movement of the cohesive sheet is governed by distinct [8].

*Category 2: Nerve Growth*

The use of ES in neural tissue engineering is a promising therapeutic approach [34]. ES positively affects four major cell types involved in peripheral nerve healing: neurones, endothelial cells, macrophages, and Schwann cells [34]. ES promotes more rapid neurite extension, cellular alignment, and cell phenotypic modifications linked with enhanced regeneration and functional recovery. Materials including hydrogel-based electrically conductive nerve guidance conduits (NGCs) can bridge gaps in the peripheral nervous system and further enhance nerve generation [35]. In vitro, the enhanced electrical conductivity of the hydrogel plays a vital role in the extension of dorsal root ganglion (DRG) axons [36]. Therefore, any environment that provides ES can alter cellular polarisation, hence regulating and enhancing cell activity in the nervous system towards the repair of the damaged cells. This can result in improvement of stretching muscles and reduction in spasticity, improvement in gait, balance, and prevention of muscle atrophy [12][15][18][21][26][37]. In practice, applying ES reduces pain score after orthopaedic surgeries [38].

*Category 3: Cellular Differentiation*

Chemical gradients, gene regulatory networks, and endogenous ion fluxes (bioelectricity) are the three major regulators of cellular activity [39]. Bioelectric signals provide crucial information on early structural growth and the restoration of normal patterns following damage [39]. Decoding bioelectric signals can change gene expression and even change genetic patterns [40]. 

Bioelectrical networks can process morphogenetic information that affects gene expression, allowing cell collectives to make large-scale growth and shape decisions [41]. All tissues have ion channel and gap junction-generated membrane potentials creating bioelectrical networking [41]. Altering bioelectric circuits through channels can effectively govern cellular collectives’ work toward appropriate changes. For instance, applying bioelectricity to the foot and ankle area improves diabetic arteriopathies, as ES can change vascular development. This will minimise chronic pain through enhancement of the arterial, and venous and lymphatic-flow [19].

## Discussion

*Summary of Evidence*

The current review assessed several applications of cell polarity in foot and ankle problems. This study consisted of 27 papers that addressed the influence of bioelectricity on wound-healing, neural development, and cellular differentiation. It is well known that the ion gradient across cell and organelle semipermeable membranes drives electrical signalling. This electrical signalling produces the electrical potential of the cell’s plasma membrane at rest, called Vmem [1]. Vmem is mainly determined by the K+ and Na+ concentrations on both sides of the membrane. Diverse ion channels, fluctuating expression of channels and isoforms with different response properties and ion affinities, and post-translational modifications of channels maintain both steady-state Vmem and dynamic responses to environmental and other stimuli. Accordingly, Vmem varies considerably during different phases, such as cellular proliferation, resting membrane potential, and developmental potential [4]. The relationship between membrane polarisation and proliferation phases have been established [4]. Accordingly, various cell types exhibit varying degrees of polarisation. For instance, metastatic cancer cells are hypopolarised, while the voltages of myoblasts and neural crest cells vary between 10 and 35 mV. Therefore, membrane polarisation and proliferative capacity are related [4]. 

Based on this connection, the research has shown that bioelectrical signalling is vital to the healing process [31]. This healing process has been discussed in three primary sections in the foot and ankle areas: regeneration and mending of the wound [8], treatment of a damaged nervous system [14], and modification of arteriopathies through changes in the cellular proliferation or expression and pattern of genes [19]. The three main regulatory networks controlling cellular activities are chemical gradients, gene regulatory networks, and bioelectricity [4]. Connections exist in these networks. For instance, bioelectric signals dictate early structural development and robust pattern restoration following an injury [4]. Chemical gradients can modify bioelectricity through ion fluxes, and bioelectric impulses can change the expression and patterns of genes by producing cell polarities and endogenous EFs [42]. Enforcing a different ES to the cells, EFs can be modified and controlled or cell function will be enhanced [42]. Changes in bioelectrical networks may also interpret morphogenetic information that affects gene expression and enable cell collectives to make large-scale decisions regarding development and shape [42].

Hence, among the three major regulatory networks, bioelectrical changes may have the greatest effect on cell activity and can regulate a large number of diseases in the foot and ankle. Moreover, applying ES or modifying the chemical gradients [16] can modify cell polarity. Therefore, understanding cell polarity and its possible roles can help practitioners find more effective ways to treat and prevent diseases[16]. For a specific disease, practitioners have access to multiple levels of symptom management. However, symptom management is not necessarily a treatment for the underlying causes. For instance, in the case of painful calf cramps, a practitioner may focus on pain treatment using analgesics, muscle relaxants, muscle stretching, calcium, and magnesium or address potential risks for calcium and magnesium deficiency, including malabsorption, wasting, or over-demand for these minerals [43]. Some measures, including analgesics, can relieve patient discomfort but usually cannot prevent predisposing factors and sources of pain. However, it is generally preferable to be aware of predisposing factors and healing obstructors, as well as their proportional effects on the current disease. Developing a comprehensive treatment plan involves assessing the risk and benefits of treating variables (intrinsic and extrinsic) while monitoring symptom improvement [44]. These predisposing variables in the case of calf muscle cramps may include hormonal changes, trauma, tension, and electrolyte imbalance [43]. The issue with this method of addressing predisposing variables is that the assessment of these variables and their potential impact on the symptoms cannot always be validated [45]. The author argues that measuring bioelectricity and cell polarity can help validate in vitro variable effects. For instance, in calf spasms, different variables can alter the bioelectricity of sarcomeres from normal. Therefore, their capacity to absorb nutrients, including calcium and magnesium, will change and this can produce muscle spasms in the final stage [45]. Achilles tendonitis is another example, inflammation of surrounding the tendon cells and perhaps their insertion into the calcaneus [46]. In this scenario, the authors declare that several intrinsic and extrinsic factors including the strain from a short calf muscle, might alter the cell polarity in the region. This alteration could be towards the polarity that absorbs more white blood cells or calcium and fibroblasts, processing inflammation, tendon injury, and fibrosis.

Bunion development is yet another example that can occur due to rotational alterations in the first metatarsal bone, or expansion of the first metatarsal bone’s head. Several genetic or acquired hazards may be involved in the development of bunions [47]. However, the author argues that real expansion in the head of the 1st metatarsal bone is impossible without alterations in the cell polarity in that location. Changes in the bioelectric and cell polarity can boost the absorption of minerals and allow the tissue to overcome mechanisms that try to control excessive development in the metatarsal bone [1]. Alternately, ion alterations and cell polarity changes in the surrounding muscles of some individuals can lead to muscular imbalance and changes in the anatomic position of the first metatarsal, including its rotation. These changes are classified as bunion [48]. Cell polarity and bioelectricity at the cellular level can play a role in the ageing process. Aging is often characterised by widespread muscle loss and cognitive impairments. During the ageing process, there is a steady decline in physical and mental function, an increase in the risk of disease, inflammatory conditions, and ultimately increased mortality [49]. In the elderly, muscles are wasted in the presence of protein, and there is the possibility of protein malabsorption at the cellular level. This malabsorption may be caused by physical and social environments or personal characteristics such as hormonal shifts, inflammation, heredity, and environmental and social variables, which vary from individual to individual [49]. 

The author argues that any of the above intrinsic or extrinsic variables will have an effect on ageing based on the amount of direct or indirect alterations they can impose on cellular polarity. For example, a psychological condition can modify cellular polarity through both humeral changes in the body and electric changes in the nerve terminals. Although these fundamental factors cannot always be studied, it is easier and more practical to measure changes in cell polarity. Additionally, changes in cell polarity and bioelectricity can influence the ageing process independently of genetics and other risk factors. By restoring normal cell polarity, inflammatory cells will be less attracted to other tissues, reducing age-related inflammatory changes [1]. 

Onychomycosis is an intriguing example of a fungal infection that affects the toenails, and it is usually treated with antifungals. This type of infection is contagious and can easily spread to other toenails. Various factors such as family history, advancing age, poor health, previous injuries, warm environment, participation in fitness activities, immunosuppressive drugs or diseases (e.g., HIV), communal bathing, occlusive footwear, and decreased blood flow can predispose individuals to this condition [50].

Determining the spread pattern of onychomycosis on a single foot can be challenging. For instance, it can be difficult to understand why a nearby toenail with more predisposing factors is healthy, while a distant toenail with fewer predisposing elements is infected [50]. The author argues that predisposing variables can alter the cellular polarity of toenail cells, making certain toenails more susceptible to fungal infection. Thus, predisposing factors are only important when they can change the cell polarity and attract the fungus agent due to the new cell polarity. 

Overall, to develop effective treatment approaches for symptomatic individuals, it is essential to create a treatment plan considering all potential predisposing variables and healing impediments [44]. Evaluating and individualizing treatment options for each patient is necessary. Using accurate instruments evaluating cell polarity and bioelectricity can help validate the probable influence of these variables. Additionally, it is possible to bypass these risk factors by adjusting the bioelectricity, directly. Of the three primary networks that govern cell activity and growth - chemical gradients, bioelectricity, and genetics - changes in bioelectricity and cell polarity are the most efficient and useful [1]. Therefore, understanding the potential involvement of bioelectricity and cell polarity in the pathophysiology of most diseases is important to develop more effective treatments that concentrate on altering cell polarity [1].

*Clinical Relevance*

Cell polarity plays an essential role in several aspects of clinical practice, including disease prevention and treatment. Practitioners need a thorough treatment plan that considers all relevant aspects and causes and compares the risk-benefit of addressing them to basic symptom control to treat diseases effectively [2]. The authors claim that it is possible to examine the impact of different variables on bioelectricity to develop an efficient treatment approach. The polarity of the adequate number of cells can play a role in attracting or repelling microbes, inflammatory cells, fibroblasts, ions, vitamins, and minerals [2]. This approach can help doctors and researchers design successful future investigations and treatment options based on changes in cell polarity. 

*Limitations*

According to the author's understanding, this is the first study to comprehensively cover the clinical implications of cell polarity in foot and ankle illnesses. However, there are concerns that certain studies may have been omitted from this review. In addition, since measuring instruments for cell polarity and bioelectricity are not always precise, it is likely that some sections of the present research may change as advancements are made [51]. The relationship between Cell Vmem and proliferative potentials is confusing, as there is no clear functional link between Vmem and the cell cycle [52]. Therefore, this aspect requires further investigation. This article only explores electrical energy as it relates to foot and ankle diseases, while our bodies utilize several other types of energy (chemical, mechanical, and electromagnetic) [42]. Although the relationship between electrical and magnetic energies, or chemical energy and the production of electrical energy, is briefly discussed, more research is necessary to determine how different types of energy affect one another and their symptoms. Moreover, while most available research focuses on the symptoms of various diseases and the effects of different medications on those symptoms, these studies often fail to evaluate potential underlying variables and associated factors. Therefore, based on the current dearth of literature, it is premature to consider cell polarity as a combining point for all predisposing factors that have yet to be adequately investigated. Several studies have shown that the use of devices for neuromuscular electric stimulation (NMES), electric stimulation (ES), or devices for ES of the peroneal nerve were ineffective in suppressing muscular spasms [12][25][29]. However, these papers were based on trial and error, and it is unclear if the use of such devices may alter the bioelectric. In other words, neither bioelectric nor cell polarities were measured, but we know that timing and amplitude vary between devices and changes in bioelectric are sensitive to timing and amplitude of the devices [53]. In many investigations [9][10][11][12][13][14][15][16][17][18][19][20][21][54][23][24][25][26][27][28][55][56][29][37][57], ES was used to restimulate the nervous system, and its efficacy was evaluated using trial-and-error methods, without considering bioelectricity and cell polarity changes. Consequently, it is unclear if ES’s observed efficacy is the result of bioelectric effects or simply imitating a pure neural response.

## Conclusion

This review provides valuable information on treatment and prevention strategies for foot and ankle diseases. It emphasizes incorporating cell polarity and considering possible factors and relevant contributing factors to reduce the risk of recurrence or other issues in patients. However, given the lack of precision in measuring bioelectricity and the inclusion of other gradients, future studies with larger sample sizes are necessary to corroborate these findings.

## Conflict of Interest

None to declare.
